# Pleiotropic effects of sphingosine-1-phosphate signaling to control human chorionic mesenchymal stem cell physiology

**DOI:** 10.1038/cddis.2017.312

**Published:** 2017-07-13

**Authors:** Giulio Innamorati, Emanuela Fontana, Federica Steccanella, Kushal Gandhi, Giulio Bassi, Valeria Zandonà, Luca Giacomello

**Affiliations:** 1 Laboratory of Experimental Pediatric Surgery, Department of Surgical Sciences, Dentistry, Gynecology and Pediatrics, University of Verona, Verona 37134, Italy; 2 Stem Cell Research Laboratory, Department of Medicine, University of Verona, Verona 37134, Italy

## Abstract

Chorionic stem cells represent a promising opportunity for regenerative medicine. A deeper understanding of the stimuli that regulate their physiology, could lead to innovative clinical approaches. We revealed the presence of multiple sphingosine-1-phosphate (S1P) receptor isoforms in chorion-derived mesenchymal stem cells (CMSCs). Their activation simultaneously propagated from the plasma membrane through Gi and other heterotrimeric G proteins and further diverged toward extracellular-signal-regulated kinase 1/2 (ERK1/2), p38 and protein kinase D 1. At a functional level, S1P signaling inhibited CMSC migration, while promoting proliferation. Instead, a reduction of cell density was obtained when S1P was combined to treatments that increased cAMP intracellular concentration. Such surprising reduction of cell viability was relatively specific as it was not observed with stromal stem cells from bone marrow. Neither it was observed by activating analogous G proteins with bradykinin nor by inducing cell death via a cAMP-independent pathway. S1P could thus reveal novel keys to improve CMSC differentiation programs acting on cAMP concentration. Furthermore, S1P receptor agonists/antagonists could become instrumental in favoring CMSC engraftment by controlling cell motility.

A number of novel approaches for regenerative therapies based on mesenchymal stem cells (MSCs) are currently under development.^1^ Among tissues of fetal origin, placenta appears to be an untapped supply of multipotent cells.^2, 3, 4^ Collecting placenta MSCs presents minimal ethical and legal concerns and warrants high yields of precursor cells endowed of expanded plasticity, low immunogenicity and immunomodulatory properties.^3, 5^

To preserve intact these valuable properties, ideally MSC expansion and differentiation should be controlled *in vitro* by mimicking physiological stimuli as close as possible. Acting on endogenous receptors would avoid the pervasive consequences associated with chemical or genetic reprogramming, particularly the risk of generating tumors. Yet, very little is known about which receptors are populating the plasma membrane of CMSCs and their function.

Similar to Wnt, CXCL12 and other G protein-coupled receptor (GPCR) agonists that coordinate trophic niches for progenitor cells,^6, 7, 8, 9^ sphingosine-1-phosphate (S1P) is emerging as a critical coordinator of morphogenesis. Starting from the initial phases of embryonic development, S1P mediates transcriptional regulation of key targets associated with survival, proliferation and pluripotency.^10^ Afterward, S1P regulates ‘cell fate’^11^ through development^12^ and tissue remodeling. In adult life, S1P contributes to regenerate adult tissues^13, 14^ such as skeletal muscle,^13^ bone^15^ and adipose tissue,^16^ by controlling proliferation and differentiation of resident mesenchymal progenitor cells.

Under stress conditions, precise stimuli mobilize stem cells from nurturing niches to travel in blood circulation. Eventually, they become attracted to local injured tissues to repair the damage. The possibility to control the tropism of exogenously administered cell precursors represents an essential aspect to achieve realistic cell-based therapies.^17^ Once again, receptor-mediated stimuli could become of a key importance. Acting as an extracellular lymph- and serum-borne ligand, S1P released by activated platelets is a major regulator of cell trafficking. The pleiotropic action of S1P is mediated by five GPCR subtypes, formerly named EDGs as in endothelial differentiation genes.^18^ In the blood system, S1P acts with CXCL12 to guide hematopoietic stem cell circulation after they leave the bone marrow to accomplish their role in body surveillance and injury recovery.^19^ S1P can sort diametrically opposite effects, depending on the cell state. Distinct GPCR subtypes were shown decisive for activating^20^ or inhibiting^21^ lymphocyte motility, and subtype 2 resulted as inhibitory. However, the receptor profile cannot by itself predict the migratory phenotype for all cell types.^22, 23^

We addressed and verified the possibility that S1P signals across the plasma membrane of CMSCs to mitogen-activated protein kinase (MAPKs) and other kinases central to the regulation of cell proliferation, differentiation and motility. Consistently, S1P affected CMSC migration and cell density. Further analysis disclosed the complexity of S1P signaling on proliferation and resistance to pro-apoptotic treatment revealing a crosstalk with the cAMP signaling pathway.

## Results

### Isolation and culture of human MSCs

CMSCs enzymatically dissociated from the chorionic membrane of five human full-term placentae were expanded as a monolayer. Cells displayed a fibroblast-like morphology and started to proliferate steadily propagating *in vitro* after successive cycles of trypsinization.

**Figure 1:**
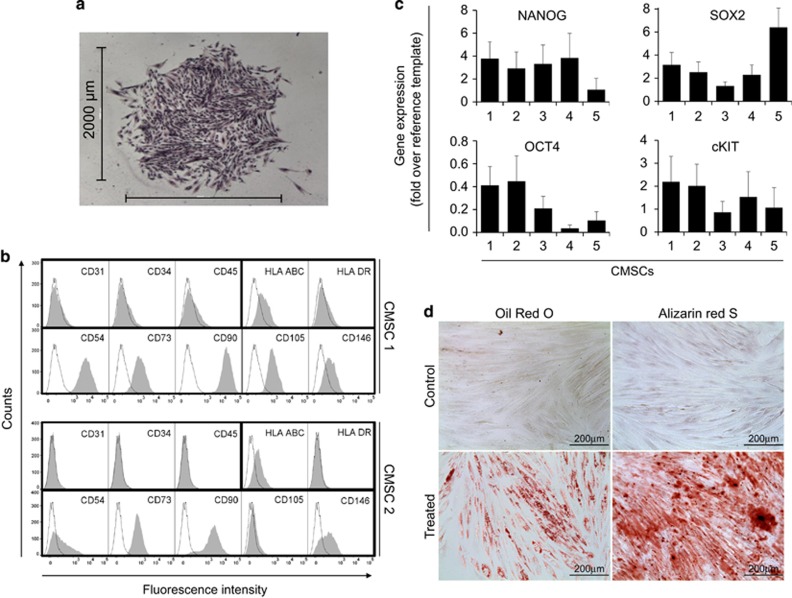
Isolation and characterization of CMSCs. Single cells in suspension were expanded adhering to culture plastic through the formation of fibroblast-like colonies. (**a**) A colony originating from a single cell, after successive cycles of amplification. Cells were fixed and stained with crystal violet. (**b**) The marker expression profile of cultured cells was analyzed by flow cytometry. The respective isotype control is shown as a dotted line. (**c**) The expression levels of transcription factors regulating multipotent properties were evaluated by RT-PCR for five preparations of CMSCs utilizing BMMSCs or Jurkat cells as a reference, *n*=5. (**d**) CMSCs were fixed after 4 weeks of treatment with the appropriate differentiation medium as indicated. Adipocytes were identified with Oil Red O to stain lipidic vacuoles in the cytosol. Osteoblasts were revealed with Alizarin red S by staining the calcium matrix in red. Staining was negative in untreated control CMSCs

Cells plated at low density formed colonies after 2 weeks (Figure 1a). Their number was counted to estimate progenitor cells and ranged from 3 to 14% of total cells seeded (Table 1).

The immunological phenotype was analyzed by flow cytometry after six passages of subculturing (the profiles of two preparations are shown in Figure 1b). Consistent with their origin,^4, 24, 25^ CMSCs were negative for MHC class II (HLA-DR), positive for MHC class I (HLA-ABC) and for MSC markers, that is, CD54, CD73, CD90, CD105 and CD146. Endothelial marker CD31 and hematopoietic markers CD34 and CD45 were not detected.

Supporting the immature nature of the CMSC preparation, RT-PCR revealed the expression of pluripotent markers, namely NANOG, SOX2, OCT4 and cKIT^24, 26^ (Figure 1c). The multilineage differentiation potential of CMSCs was confirmed by inducing their differentiation in adipocytes and osteoblasts (Figure 1d).

### CMSCs express S1P receptors

**Figure 2:**
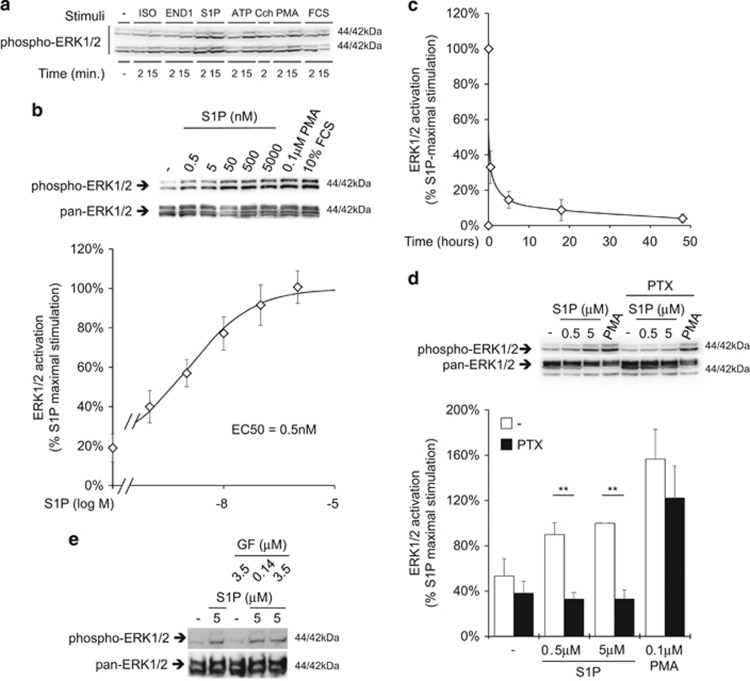
GPCRs mediated ERK1/2 activation in CMSCs. The activation state of ERK1/2 was analyzed by western blot using an antibody raised against specific activating phosphorylation sites. (**a**) CMSCs were stimulated for the indicated time with several GPCR ligands, in particular 10 *μ*M isoproterenol (ISO), 0.1 *μ*M endothelin 1 (END1), 5 *μ*M S1P, 100 *μ*M ATP and 100 *μ*M carbachol (Cch). A unit of 0.1 *μ*M PMA and 10% FCS were used as positive controls to bypass GPCRs. (**b**) The relationship between S1P concentration and ERK1/2 phosphorylation was described after 10 min of stimulation. From the fitting curve, the EC50 was estimated to be 0.5 nM, *n*=7. The panel above the graph shows a representative experiment. (**c**) Phosphorylation of ERK1/2 was measured over a 48 h time period using 5 *μ*M S1P, *n*=3. (**d**) Gi was inhibited by pre-treatment with 200 ng/ml PTX before stimulating for 5 min with S1P or PMA as indicated, **P<0.01, *n*=4. The panel above the graph shows a representative experiment. (**e**) All PKC isoforms were inhibited by pre-treatment with the indicated concentration of GF 109203X before stimulating for 5 min with S1P, *n*=3

To identify GPCRs present on CMSC plasma membrane, saturating concentrations of several ligands (for a complete list of GPCR ligands used in this study see Supplementary Table S1) were tested for their ability to activate extracellular-signal-regulated kinase 1/2 (ERK1/2) in two representative preparations. Ubiquitous purinergic and muscarinic receptors produced ERK1/2 activation in response to ATP and carbachol, respectively. S1P stimulation was higher and comparable to phorbol-12-myristate-13-acetate (PMA), the highly potent protein kinase C (PKC) activator (Figure 2a).

The activation was dose-dependent and the EC50 was estimated in a low nanomolar range, consistent with the effect being mediated via high-affinity GPCRs (Figure 2b). ERK1/2 activation was transient in time (Figure 2c) and it was completely inhibited by pertussis toxin (PTX), indicating that the pathway was fully Gi-dependent (Figure 2d). On the other hand, the inhibition of all PKC isoforms by GF 109203X produced no effect (Figure 2e).

**Figure 3:**
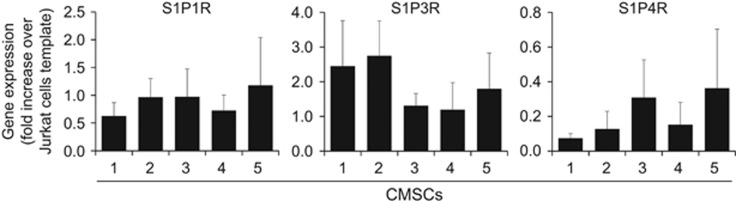
S1PR expression in CMSCs. mRNA transcription levels of the five different S1PRs were estimated by RT-PCR. mRNA prepared from Jurkat cells was used as a reference

For all five preparations tested, specific PCR primers demonstrated transcription of three out of five S1P receptor (S1PR) subtypes (Figure 3; Supplementary Figure S1). No mRNA was detected for S1P2R and S1P5R.

**Figure 4:**
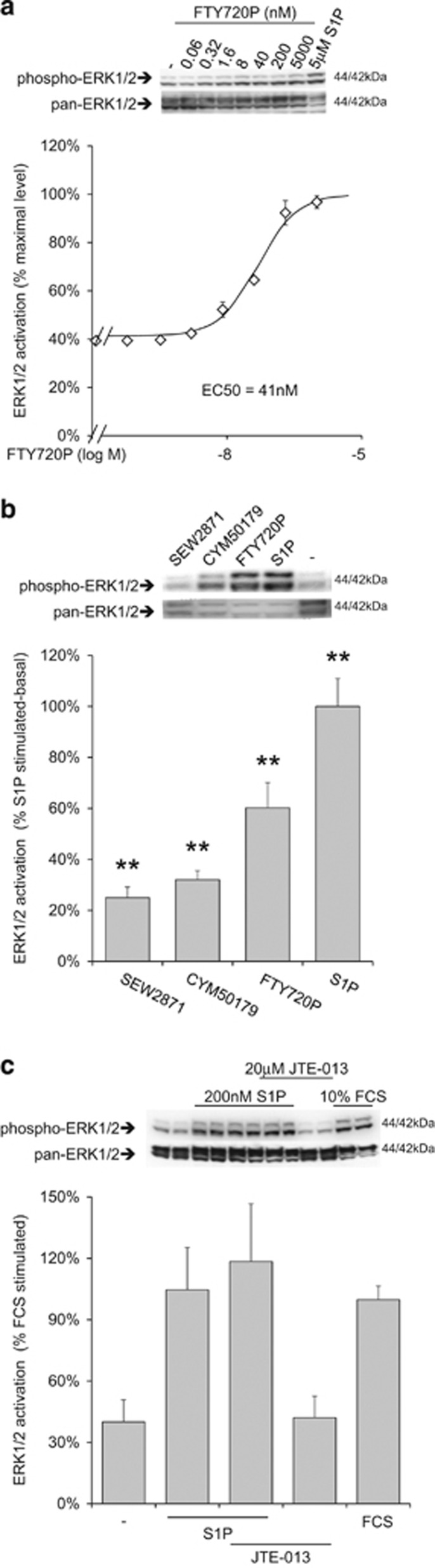
Multiple S1PR isoforms mediate ERK1/2 activation in CMSCs. (**a**) Receptor activity was assessed by western blot measuring ERK1/2 activation 10 min after stimulation with increasing concentrations of FTY720P. The EC50 was estimated to be 41 nM, *n*=3. The panel above the graph shows a representative experiment. (**b**) ERK1/2 activation was analyzed in response to selective S1PR agonists. In particular 1 *μ*M SEW2871, 1 *μ*M CYM50179, 0.5 *μ*M FTY720P and 5 *μ*M S1P; **P<0.01 *versus* basal, *n*=3. The panel above the graph shows a representative experiment. (**c**) The effect of the antagonist JTE-013 was assessed on S1P-stimulated ERK1/2 activation, *n*=3. The panel above the graph shows a representative experiment

GPCR involvement in S1P signaling was confirmed by the increase of ERK1/2 phosphorylation observed in response to FTY720P, a ligand selective for all S1PRs but S1P2R. Also in this case, the EC50 was in the nanomolar range (Figure 4a).

A preliminary screening with additional S1P analogs (Supplementary Table S1) was performed to further detail which receptor subtypes were responsible for ERK1/2 activation. SEW2871, a S1P1R selective agonist,^27^ was effective, although to a minor extent compared to S1P. CYM50179, a S1P4R selective agonist,^28^ produced a stronger stimulation (Figure 4b), while JTE-013, a S1P2R antagonist,^29^ did not prevent the effect of S1P, ruling out S1P2R (Figure 4c). All together, these results suggested a combined action of multiple S1PR subtypes converging on Gi-dependent ERK1/2 activation.

### Downstream signaling of S1PRs

**Figure 5:**
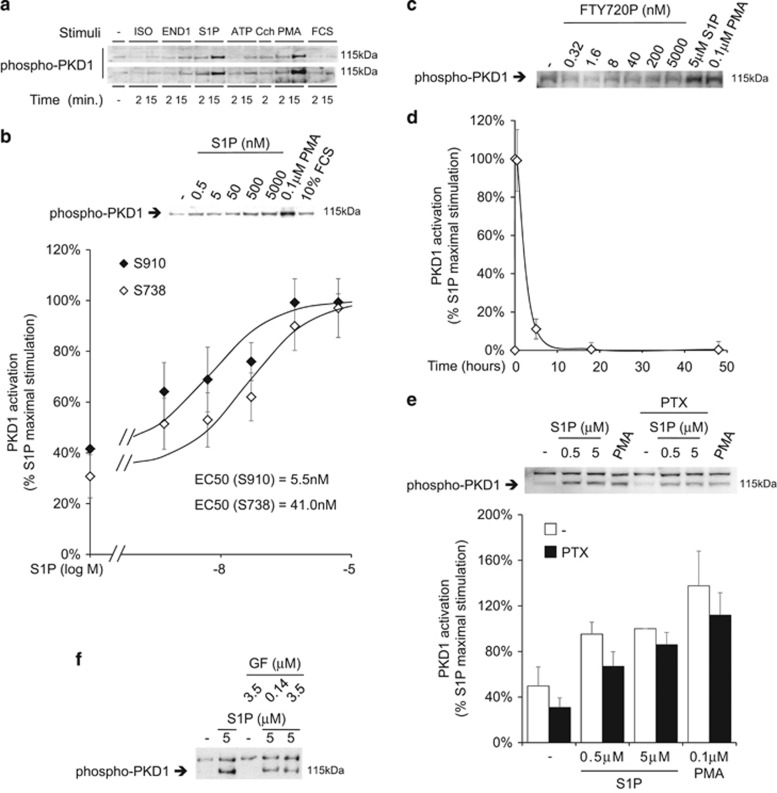
Effect of S1PR ligands on PKD1 activation in CMSCs. PKD1 activation state was analyzed by western blot using antibodies raised against specific activating phosphorylation sites. (**a**) CMSCs were stimulated with GPCR ligands as indicated and analogously to Figure 2. (**b**) The relationship between S1P concentration and PKD1 phosphorylation was analyzed 10 min after stimulation. Two distinct phosphorylation sites were considered. On the basis of the fitting curves, the estimated EC50 is 41 nM for S738 and 5 nM for S910, *n*=7. The panel above the graph shows a representative experiment. (**c**) FTY720P effect on PKD1 phosphorylation was assessed 10 min after stimulation with increasing concentrations of ligand, *n*=3. (**d**) PKD1 phosphorylation was measured over a 48 h time period using 5 *μ*M S1P, *n*=3. (**e**) After Gi inhibition by 200 ng/ml PTX, cells were stimulated with S1P or PMA as indicated, *n*=4. The panel above the graph shows a representative experiment. (**f**) All PKC isoforms were inhibited by pre-treatment with the indicated concentration of GF 109203X before stimulating for 5 min with S1P, *n*=3

S1P signaling was not limited to ERK1/2 but included protein kinase D 1 (PKD1), as it was revealed by phosphorylation of two activation-dependent sites, S738 and S910 (Figures 5a and b). Also in this case, nanomolar concentrations of either S1P or FTY720P were sufficient to promote PKD1 activation (Figures 5 b and c). In addition of being dose-dependent, the effect was transient (Figure 5d).

**Figure 6:**
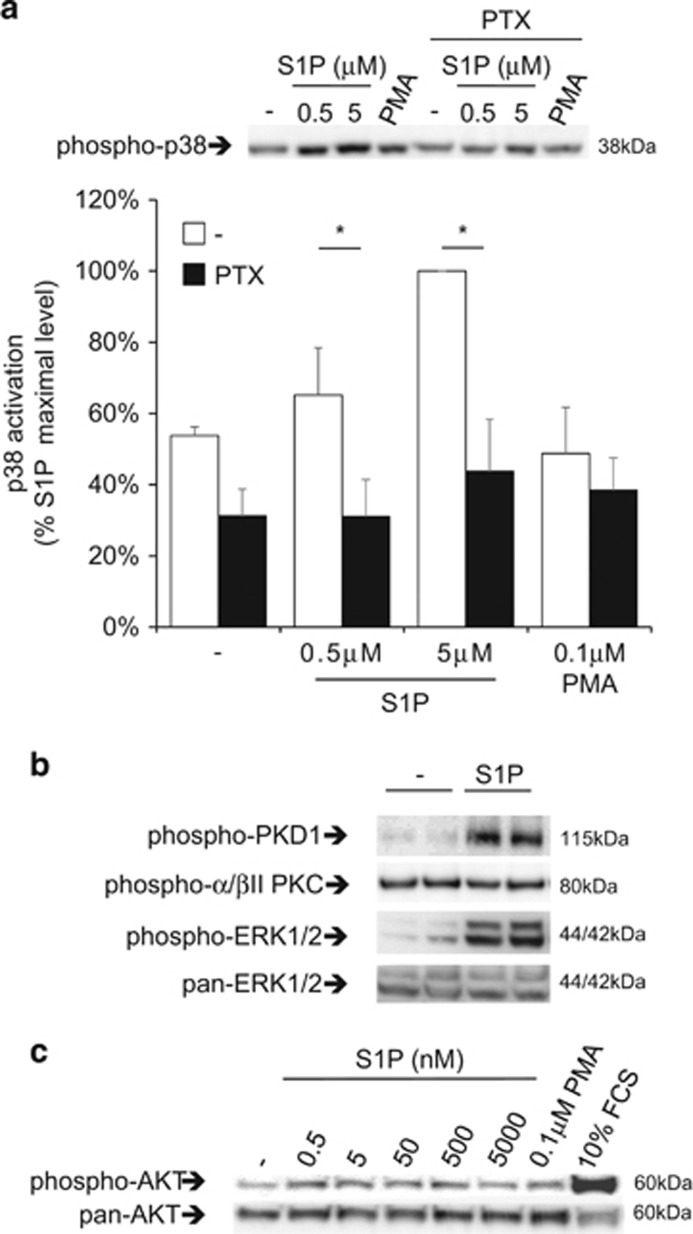
Effect of S1P on p38 and AKT activation in CMSCs. (**a**) The effect of S1P stimulation for 5 min was assessed after Gi inhibition by 200 ng/ml PTX analyzing p38 activation state by western blot, *P<0.05, *n*=3. The panel above the graph shows a representative experiment. (**b**) The activation state of PKC isoforms was analyzed by western blot after treatment with 5 *μ*M S1P, *n*=3. (**c**) AKT activation state was analyzed after treatment with S1P at different concentrations by western blot. *n*=7. A representative experiment is shown

Similar results were obtained while analyzing p38 activation, which appeared Gi-mediated like for ERK1/2 (Figure 6a).

Opposite to MAPK activation, agonist-dependent activation of PKD1 was unaffected by PTX (Figure 5e) but it was sensitive to GF 109203X (Figure 5f). The latter result suggests the involvement of Gq/11 or G12/13, and PKC family members despite we observed no activation of classic PKC isoforms (Figure 6b).

AKT phosphorylation remained unaltered after S1P exposure. It thus appears that in CMSCs, S1P downstream signaling does not involve the PI3K pathway (Figure 6c), but activates multiple kinases instrumental to a large variety of biological functions.

### Functional consequences of S1P signaling in CMSCs

S1P influences several aspects of morphogenesis, such as cell growth, collective cell migration and tissue inductive events.^12^ We sought functional assays aimed to analyze proliferation, migration and differentiation.

Cells were plated at low density and their expansion was evaluated after 7 days in the presence of fetal calf serum (FCS) that was charcoal-stripped to remove S1P possibly released by aggregating calf platelets.^30^ Addition of S1P increased cell density (Table 2), the increase was relatively contained, but significant at both S1P concentrations tested.

**Figure 7:**
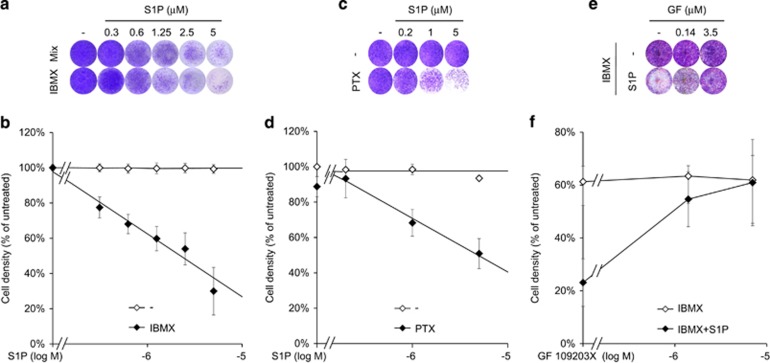
Functional consequences of combined treatment of S1P and cAMP modulators on CMSCs. (**a**) CMSCs were fixed and stained with crystal violet after 3 weeks in the presence of 1 mM IBMX, alone or in combination with the other components of the adipose differentiation cocktail (mix). S1P was added at the indicated concentration for the entire period. (**b**) Increasing concentrations of S1P were combined to a fixed amount of IBMX (1 mM), *n*=5. (**c**) After pre-treatment with 200 ng/ml PTX, CMSCs were stimulated with increasing concentrations of S1P. The next day, cells were fixed and stained with crystal violet. (**d**) The experiment was repeated and the effect was quantified, *n*=8. (**e**) The toxicity produced by combining 5 *μ*M S1P to 1 mM IBMX was assessed after pre-treatment with increasing concentrations of GF 109203X. (**f**) The experiment was repeated and the effect was quantified, *n*=4

The positive effect on total cell number was reversed when S1P was combined with isobutyl-1-methylxantine (IBMX), added either as part of an adipocyte differentiation cocktail or separately (Figure 7a). Increasing S1P concentration in a medium containing 1 mM IBMX progressively reduced cell density (Figure 7b). Charcoal-stripped FCS showed no significant difference as compared to regular FCS (Supplementary Figure S2a). According to a 3-(4,5-dimethylthiazol-2-yl)-2,5-diphenyltetrazolium bromide (MTT) assay, the effect was unlikely caused by a slight and not statistically significant reduction of cell proliferation, no matter if FCS was charcoal-stripped (not shown) or not (Supplementary Figure S2b). Most likely, the reduction of cell density was produced by cell death. Flow cytometry analysis of cell viability after 36 h in the presence of 1 mM IBMX shows that S1P produced an increase of 24% (±3%, *P*<0.01, *n*=3, [S1P]=5 *μ*M) of the population in early stages of apoptosis (annexin V-positive and propidium iodide-negative).

In alternative to inhibit phosphodiesterases with IBMX, preventing inhibitory Gi activity on adenylyl cyclase with PTX can also increase cAMP intracellular concentration. Correspondingly, PTX treatment resulted in a S1P dose-dependent reduction of cell viability (Figures 7c and d). The effect was reverted by treating cells with GF 109203X, an inhibitor of novel and atypical PKCs, like PKD1 (Figures 7e and f).

The combination S1P–IBMX did not produce the same effect on BMMSCs (Supplementary Figure S2c), and interestingly, no S1P effect was observed co-administering gemcitabine, a pro-apoptotic stimulus acting independently from cAMP (Supplementary Figure S2d).

Similar to S1P, bradykinin stimulates Gi and Gq/11 via GPCR^31^ and, on turn, activates ERK1/2 to a comparable level (Supplementary Figure S2e). However, the pro-apoptotic effect of IBMX could not be amplified by bradykinin (Supplementary Figure S2f).

Altogether, these results indicate a certain degree of specificity for the crosstalk occurring between S1P and cAMP. In other words, in CMSCs the concentration of cAMP appears to be a switch that converts the effect of S1P from tonic to pro-apoptotic.

The marked consequences on cell viability imposed to reduce the concentration of IBMX to 0.5 mM for a meaningful analysis of S1P effect toward adipocyte differentiation,^32, 33^ under these conditions no significant difference was observed (Supplementary Figure S3a). Similarly, S1P had no effect on osteogenesis (Supplementary Figure S3b). Cell differentiation was hindered by charcoal-stripping of FCS but the effect could not be ascribed to S1P as replenishing the phospholipid (5 *μ*M) did not restore osteogenesis (not shown).

MSC have been repeatedly reported for their immunomodulatory properties. CMSCs are not an exception and were shown to inhibit T-cell proliferation.^34^ We confirmed this finding, however, this important property was not affected by S1P (Supplementary Figure S3c).

**Figure 8:**
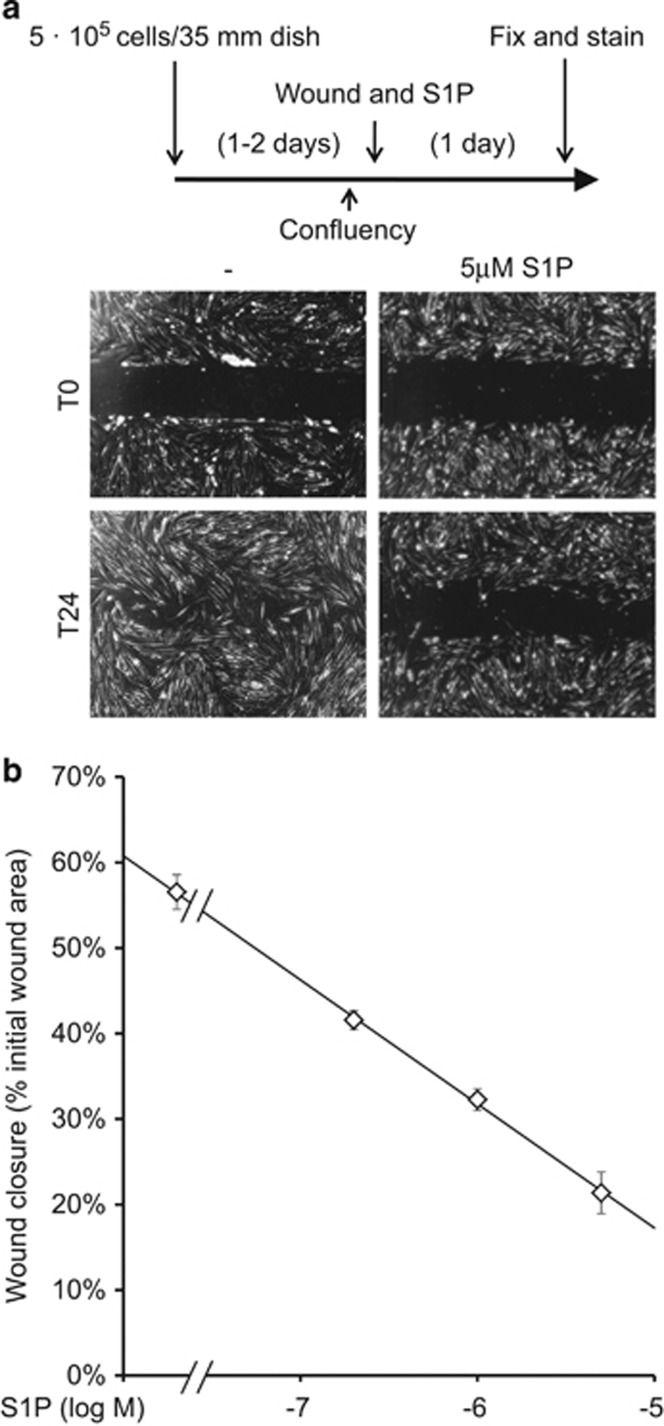
Functional consequences of S1P signaling on CMSC motility. (**a**) Wound-healing assay was performed after seeding CMSCs at confluency. Cells were treated for 24 h with S1P in 0.3% FCS as indicated in the scheme. Below, representative pictures of initial scratches (T0) and of the same field after 24 h treatment (T24). (**b**) The area invaded by the cells at T24 was quantified in respect to the initial size of the wound and plotted *versus* increasing concentrations of S1P. Each experiment considered the wound surface of more than 50 fields for data point, *n*=3

No evident effect of S1P was observed in wound-healing assays measuring the time required by the cells to fill the gap in the presence of 2% bovine serum albumin (BSA) or 10% FCS (Supplementary Figures S4a and b). However, utilizing a medium supplemented with 0.3% FCS, S1P markedly inhibited cell migration in a dose-dependent manner (Figure 8; Supplementary Video S5). Spontaneous migration was not reduced by charcoal-stripping (data not shown). Under analogous conditions, single cells were tracked by time-lapse microscopy. The effect of S1P was evident after 5 h treatment determining a reduction of cell velocity from 5.3 to 2.3 *μ*m/h (Supplementary Figure S4c).

## Discussion

Several studies isolated and differentiated MSCs from human term placenta.^24, 35, 36^ However, CMSCs remain poorly characterized when compared to MSCs obtained from bone marrow or other sources. A better knowledge about their receptor expression profile could deliver crucial tools to manipulate them, while fully preserving their safety (see introduction). S1P is reported upstream several pleiotropic regulators of cell physiology and as a consequence of that, as a crucial regulator of cellular processes such as proliferation, migration survival and differentiation.^37^

We provide important indications in this sense by demonstrating that the exposure to S1P selectively stimulates endogenous PKD1, while leaving classic PKC isoforms surprisingly unaffected. PKD1 is a key regulator of multiple functions, including cell polarity, proliferation, migration and differentiation.^38^ S1P was previously reported for modulating MAPK activation in other cellular systems.^39, 40^ In CMSCs we observed a parallel transient increase of ERK1/2 and p38 phosphorylation operated by S1P via a Gi-dependent signaling branch that diverges from the PTX insensitive branch oriented on PKD1.

The human genome encodes for five distinct S1PRs. Such diversified coupling could be responsible for splitting the signal. Numbered 1–5,^37^ S1P1R, S1P2R and S1P3R are widely expressed while S1P5R is prevalently expressed in the central nervous system and S1P4R in hematopoietic cells,^41^ Schwann cells^42^ and other precursor cells. As compared to S1P, its analogs promoted partial ERK1/2 activation in CMSCs. An incomplete response could be a consequence of reduced potency, or more likely of selective binding discriminating among receptor subtypes.^43^ Significant ERK1/2 activation was observed in response to S1P1R specific agonist, SEW2871. S1P2R is reported as poorly coupled to Gi and ERK1/2,^44^ and its mRNA could not be detected. Consistently, its antagonist JTE-013 was ineffective. S1P3R and S1P4R mRNAs were also present and indeed FTY720P produced an intermediate activation level, only second to S1P itself.

Altogether, our data portray S1P triggering a signaling circuitry that branches already at the level of the plasma membrane of CMSCs.

In muscular,^13^ neural^45^ and hepatic^46^ tissues S1P was shown to support potency, proliferation and self-renewal of cellular precursors. In CMSCs, we discovered that the sphingolipid increases cell density if administered by itself, but unexpectedly, it markedly impacts cells viability if combined with either IBMX or PTX. The ‘switch’ sorting between the two opposite effects is most likely related to the consequent increase of cAMP intracellular concentration. In addition, the pro-apoptotic signaling branch is presumably MAPK-independent as the S1P activation of ERK1/2 and p38 is assumed fully blunted by the pre-treatment with PTX (Figures 2 and 6). On the other side, the pro-apoptotic co-signal appeared to be driven by a PKC-dependent branch because the outcome was reverted by GF 109203X, a selective PKC inhibitor that does not discriminate for the different isoforms.

The intracellular level of cAMP appears to be determinant for driving commitment of MSCs.^47^ S1P is widely reported as an anti-apoptotic agent, the overturn provoked by the crosstalk might have implications for differentiation protocols combining high FCS concentrations to IBMX. In fact, the latter is a phosphodiesterase inhibitor that is used for inducing neurogenesis^48^ or adipogenesis.^49^

In maturing 3T3-L1 preadipocytes, S1P significantly decreased lipid accumulation.^50^ In C3H10T1/2 stem cell line, S1P stimulation skewed differentiation from adipogenic to osteogenic.^47^ We did not observe a significant effect on osteogenic nor adipogenic differentiation. A possible explanation for the variety of effects produced by S1P on differentiation is that the S1PR expression profile may evolve during the process. Ongoing efforts are being dedicated to better correlate *in vitro* S1P signaling to the commitment toward other lineages. Yet, a more significant analysis may require moving to animal models, where FTY720 was demonstrated to possess anti-obesity properties.^51^

*In vivo* maturation is often contingent to migratory processes concurring to controlled cell proliferation.

Serum S1P levels are relatively high comparing to peripheral tissues.^52^ The resulting gradient can be either chemoattractive or chemorepulsive depending on the subtype of receptor expressed.^53^ We observed a dose-dependent inhibition of CMSC motility in response to S1P.

Chemokine-guided stem cell recruitment is considered as a fundamental step toward *in situ* tissue engineering,^54^ and additional chemotactic stimuli could result as equally valuable. Our results warrant the rationale for studying the activity of S1PRs in directing tissue tropism of CMSCs *in vivo*. Experiments with CMSCs will be designed to reproduce analogous studies that demonstrated S1P-mediated homing of hematopoietic stem cells at sites of tissue injury.^55^

Activated platelets represent the major source of extracellular S1P. Monocytes and vascular cells may also contribute to an increase of local concentration, linking the coagulation system to inflammatory responses.^56^ On the other side, FTY720 was proposed as a surrogate anti-inflammatory chemokine capable of conditioning local tissues with angiocrine factors like CXCL12 and preferentially recruit anti-inflammatory monocytes.^57^ Hence, opportune orchestration of S1PR activity mediated by agonists/antagonists could offer a number of synergistic effects instrumental, not only to prevent the egression of transplanted cells but also to enhance healing outcomes, tissue regeneration and biomaterial implant functionality.

## Conclusion

We demonstrated that S1P represents an important modulator of CMSC physiology.

By interacting with selected receptor subtypes coupled to different G proteins, S1P produced multiple effects ranging from the activation of PKD1 and MAPK signaling to more indirect functions, such as controlling cell motility and balancing cells death *versus* proliferation.

## Materials and Methods

### Cells isolation and culture

Following informed consent (approved by the Ethical Committee of the Azienda Ospedaliera Universitaria Integrata di Verona, no. 0054, 4 June 2012) five human term placentae were collected after cesarean section and treated in accordance with approved guidelines. CMSCs were expanded as previously described by Soncini *et al.*^58^ Chorion fragments were collected and washed twice in physiologic saline solution supplemented with 100 U/ml penicillin and 100 *μ*g/ml streptomicin. Dissociation was achieved by mechanical digestion using a scalpel followed by sequential enzymatic digestions: 24 U/ml dispase II for 5 min; 0.75 *μ*g/ml collagenase for 30 min; and 0.25% trypsin for 5 min repeated three times.

Each enzymatic digestion was in phosphate-buffered saline (PBS) supplemented with 20 *μ*g/ml DNase I at 37 °C and was blocked in Dulbecco’s modified Eagle’s medium (DMEM) high glucose supplemented with 10% FCS, 1% penicillin/streptomycin and 1% l-glutamine (complete medium). Cells were recovered each time by centrifugation for 5 min at 400 × *g*. Finally, cells in suspension were filtered through a 100 *μ*m cell strainer, plated in complete medium and expanded. Seventy or eighty percent confluency was reached after 7 days. All reagents were from Sigma-Aldrich (St. Louis, MI, USA).

Human MSCs were isolated from bone marrow (BMMSCs) aspirates of healthy donors in ‘accordance’ with the guidelines approved by the Ethical Committee of the Azienda Ospedaliera Universitaria Integrata di Verona, no. 1828, 12 May 2010. Cells were cultured in alpha-minimal essential medium, 10% FCS, 100 U/ml penicillin and 100 mg/ml streptomycin (all from Gibco, Grand Island, NY, USA). After 72 h, nonadherent cells were removed and the medium was replaced twice a week.^59^

### CMSC characterization

To assess the expression of different markers, CMSCs were labeled with the following monoclonal antibodies: IgG1*κ*-PE; CD31-PE; CD34-PE; CD45-PE; CD73-PE; CD90-PE; CD105-PE; CD54-PE; CD146-PE; and HLA-ABC-PE (BD Biosciences, Franklin Lakes, NJ, USA); IgG1*κ*-FITC; and HLA-DR-FITC (Beckman Coulter, Brea, CA, USA).

A total of 10^5^ CMSCs per tube were incubated with the selected monoclonal antibody or appropriate isotype control in PBS for 15 min at room temperature and, after one wash and the addition of TO-PRO-3 (Life Technologies, Wilmington, DE, USA), samples were analyzed by flow cytometry using FACSCanto II (BD Biosciences).

Total RNA was isolated from CMSCs, BMMSCs or Jurkat cells using RNAeasy mini kit (Qiagen, Hilden, Germany) according to the manufacturer’s instructions. Purified RNA was quantified with Nanodrop spectrophotometer (Life Technologies) and complementary cDNA was generated from 2 *μ*g of RNA using random primers by high-capacity cDNA reverse transcription kit (Applied Biosystems, Foster City, CA, USA). Gene expression was quantified on a ABI Prism 7300 (Applied Biosystems) using the SYBR green method^60^ and utilizing predesigned primers (KiCqStart SYBR Green Primers by Sigma-Aldrich).

Quantitative values were obtained from the threshold cycle value (Ct). Each sample was normalized on the basis of its GAPDH content. PCR products were visualized by agarose gel electrophoresis.

### Cell differentiation

CMSC differentiation potential was assessed in 96-well plates by testing their ability to differentiate into adipocytes and osteoblasts in the presence of specific differentiation media.^61^ Adipogenic differentiation was assessed after 4 weeks of culture in DMEM high glucose containing 10% FCS, 1*μ*M dexamethasone, 10 *μ*g/ml insulin, 60 mM indomethacin and 1 mM IBMX that was reduced to 0.5 mM in the experiments combining S1P. Osteogenic differentiation was assessed after 3 weeks in DMEM low glucose containing 5% FCS, 0.1 *μ*M dexamethasone, 0.15 mM ascorbic acid and 2 mM *β*-glycerophosphate. The differentiation media were replaced twice a week. Oil Red O and Alizarin red S dyes were used to identify adipocytes and osteoblasts, respectively. When combined to S1P, differentiation was achieved using either regular or charcoal-stripped FCS prepared according to Obinata.^30^ All reagents were from Sigma-Aldrich.

### Cell treatments

GPCR ligands were bought from Sigma-Aldrich, solubilized according to the manufacturer’s instructions and administered in the growth medium supplemented either with FCS or BSA, as specified in the text.

For experiments requiring pre-treatment, before stimulation CMSCs were exposed to GF 109203X (Sigma-Aldrich) or PTX (Sigma-Aldrich), for 1 or 2 h, respectively.

### Cell expansion and toxicity assays

CMSCs were seeded in 96-well plates at a density of 10^3^ cells/well or 7 × 10^3^ cells/well for proliferation and toxicity assays, respectively. After 12 h, S1P or bradykinin was added alone or in combination with IBMX, PTX, GF 109203X or gemcitabine. DMEM was supplemented with regular or charcoal-stripped^30^ FCS when specified in the text. Half of the volume was replaced on the fourth and seventh days. On the tenth day, nonadherent cells were withdrawn by gentle washing with PBS. Adherent cells were fixed with 4% paraformaldehyde and stained for 10 min in 5 mg/ml crystal violet in 2% ethanol. In all cases, a picture of the entire bottom of each well was obtained at the end of the experiment utilizing a scanner (Nanogen Advantage Diagnostic, Strassberg, Germany). Cell density was quantified utilizing a routine of ImageJ 1.46r software (National Institute of Health, Bethesda, MD, USA).

### Apoptosis assay

Confluent cells in 12-well plates were treated with 1 mM IBMX and 5 *μ*M S1P, alone or combined, in DMEM 10% regular FCS at 37 °C under 5% CO_2_ for 36 h., The apoptotic rates of CMSCs were determined by annexin V detection kit (BD Biosciences), according to the manufacturer’s instructions and analyzed by flow cytometry.

### MTT proliferation assay (referee 1, minor point 2)

The MTT assay (CellTiter 96, Promega, Madison, WI, USA) was performed to assess cell proliferation. A total of 5 × 10^3^ CMSCs per well seeded into 96-well plates were stimulated with the indicated treatments. After 24 h, MTT solution was added to each well and the plates were incubated for 1 h, according to the manufacturer’s instructions. The absorbance was measured at 570 nm using a spectrophotometer (VICTOR Multilabel Plate Reader, PerkinElmer, Waltham, MA, USA). The absorbance was normalized by the number of cells, that is, the number of nuclei stained with Hoechst 3342 (1:5000 dilution; Invitrogen, Carlsbad, CA, USA).

### Western blot analysis

For analysis of ERK1/2, p38, PKD1, PKCs *α*/*β*II and AKT, cells were grown to confluency and starved for 3 days in 2% BSA DMEM. Agonist stimulation was achieved in the same medium treating for the indicated time. At the end, cells were washed briefly with ice-cold PBS, lysed in 2 × SDS-PAGE sample buffer, collected in microfuge tubes and stored at −20 °C. Approximately 20 *μ*g of proteins were separated on 10% SDS-PAGE gels under reducing conditions, then blotted to polyvinylidene difluoride membranes. Blocking (1 h) and subsequent overnight incubation was in Tris-buffered saline (TBS) containing 0.5% Tween-20 (t-TBS) supplemented with 5% dry fat-free milk.

Membranes were probed with antibodies to detect PKD1 phosphorylated on S738, S910 and total (1 *μ*g/ml Sigma-Aldrich SAB-4300060, SAB-4300075 and SAB-4502371, respectively), ERK1/2 phosphorylated and total (0.5 *μ*g/ml Sigma-Aldrich M7802 and M5670, respectively), p38 phosphorylated on T180, Y182 and total (1 *μ*g/ml Cell Signaling (Leiden, The Netherlands) 9211 and 9212, respectively), PKC*α*/*β* II phosphorylated (1 *μ*g/ml Cell Signaling 9375), and AKT phosphorylated and total (1 *μ*g/ml Cell Signaling 4058S and 4691, respectively) all in 5% milk in t-TBS for 1 h. After washing in fresh t-TBS, membranes were incubated with horseradish peroxidase (HRP)-conjugated anti-rabbit or anti-mouse secondary antibody at 1:10 000 in 5% milk in t-TBS for 1 h, washed in fresh t-TBS (twice for a total of 20 min) and the bands were detected using Luminata Forte Western HRP Substrate (Millipore, Billerica, MA, USA) and Syngene G Box (Syngene, Cambridge, UK). Band intensities were quantified using ImageJ 1.46r software.

### Cell migration assay

Cell motility was assessed by a scratch assay. CMSCs were seeded in 35 mm dishes, grown in DMEM 10% FCS incubated at 37 °C in 5% CO_2_ to create confluent monolayers. The monolayers were scratched using a sterile pipette tip. Growth medium was replaced as indicated. After 24 h, the filling of the scratch was digitally measured on at least 50 photographs for each condition by analyzing 12 scratches from 2 dishes. Photoshop software was used to measure the empty area.

To calculate cells velocity, 10^3^ cells were seeded in 96-well plate and fed with DMEM 0.3% FCS.

After being stained with Hoechst 3342 (1 : 5000 dilution), cells were treated with 5 *μ*M S1P or vehicle, and incubated at 37 °C under an automated microscope (EVOS FL Auto, Thermofisher, Waltham, MA, USA). Cell movement was tracked each 30 min. Multitracker plugin of ImageJ software was used for cell tracking analysis.

### Immunomodulation assay (referee 2, major point 4)

T-cell effectors (CD3-positive) were purified from peripheral blood using a negative selection kit (Pan T Cell Isolation Kit, Miltenyi Biotec, Bergisch Gladbach, Germany). T-cell purity after the separation (at least 95%) was assessed by flow cytometry.

CMSCs seeded at 80% confluency were stimulated or not for 72 h with 2.5 *μ*M S1P in Roswell Park Memorial Institute (RPMI) medium supplemented with 10% FBS (both from Sigma-Aldrich).

To evaluate CMSC-mediated immunomodulation of T-cell proliferation, rested and primed CMSCs were collected and seeded with T cells at either 2 × 10^4^ cells/well of a flat-bottom 96-well plate (corresponding to a confluent monolayer), or 2 × 10^3^ cells/well concentration. After CMSC adhesion, 2 × 10^5^ T cells previously stained with 5 *μ*M carboxyfluorescein succinimidyl ester (Life Technologies) were added to CMSC cells. T cells were activated with 0.5 *μ*g/ml of crosslinking anti-CD3 and anti-CD28 antibodies (Sanquin, Amsterdam, The Netherlands) for 6 days in RPMI supplemented with 10% human AB serum (EuroClone, Pero, Italy).

At the end of co-culture, cells were detached by trypsin and stained with anti-human CD45 APC-eFluor 780 (eBiosciences, San Diego, CA, USA) and TO-PRO-3 iodide. The proliferation was assessed on viable (TO-PRO-3-negative and CD45-positive) T cells by flow cytometry and expressed as the percentage of cells undergoing at least one cell division. The proliferation rate was obtained according to the following formula: (CD45-positive cell proliferation with CMSCs)/(CD45-positive cell proliferation without CMSCs) × 100.

### Statistical analysis

All experiments were repeated three or more times and performed at least in duplicates. Results are reported as mean±S.E.M. Significant differences between two or more treatment groups were evaluated using Student’s *t*-test, Kruskal–Wallis test or one-way analysis of variance. When *P*<0.05, the differences were considered to be statistically significant.

## Figures and Tables

**Table 1:** Clonogenicity of CMSCs

**CMSC preparation**	**Clonogenicity**
1	14±2%
2	3±2%
3	4±1%
4	12±3%
5	4±2%

After 2 weeks of culturing, single CMSCs formed colonies (defined as 50 or more adjacent cells). Colonies were counted, divided by the number of seeded cells and expressed as %±S.D.

**Table 2:** Functional consequences of S1P signaling on CMSC density

**S1P (*****μ*****M)**	**0.25 *****μ*****M**	**1 *****μ*****M**
Density increase	21%	20%
S.E.M.	9%	10%
*P*	<0.05	<0.05
*n*	9	13

The table depicts variations in density of cultured CMSCs due to the presence of S1P
