# Supplementary material for: Pleiotropic effects of sphingosine-1-phosphate signaling to control human chorionic mesenchymal stem cell physiology
Source: Cell Death Dis. 2017 Jul 13;8(7):e2930–. doi: 10.1038/cddis.2017.312 (PMC5550859; doi:10.1038/cddis.2017.312)
Supplement: Supplementary Figure S1 [file cddis2017312x1.ppt]

## Slide 1
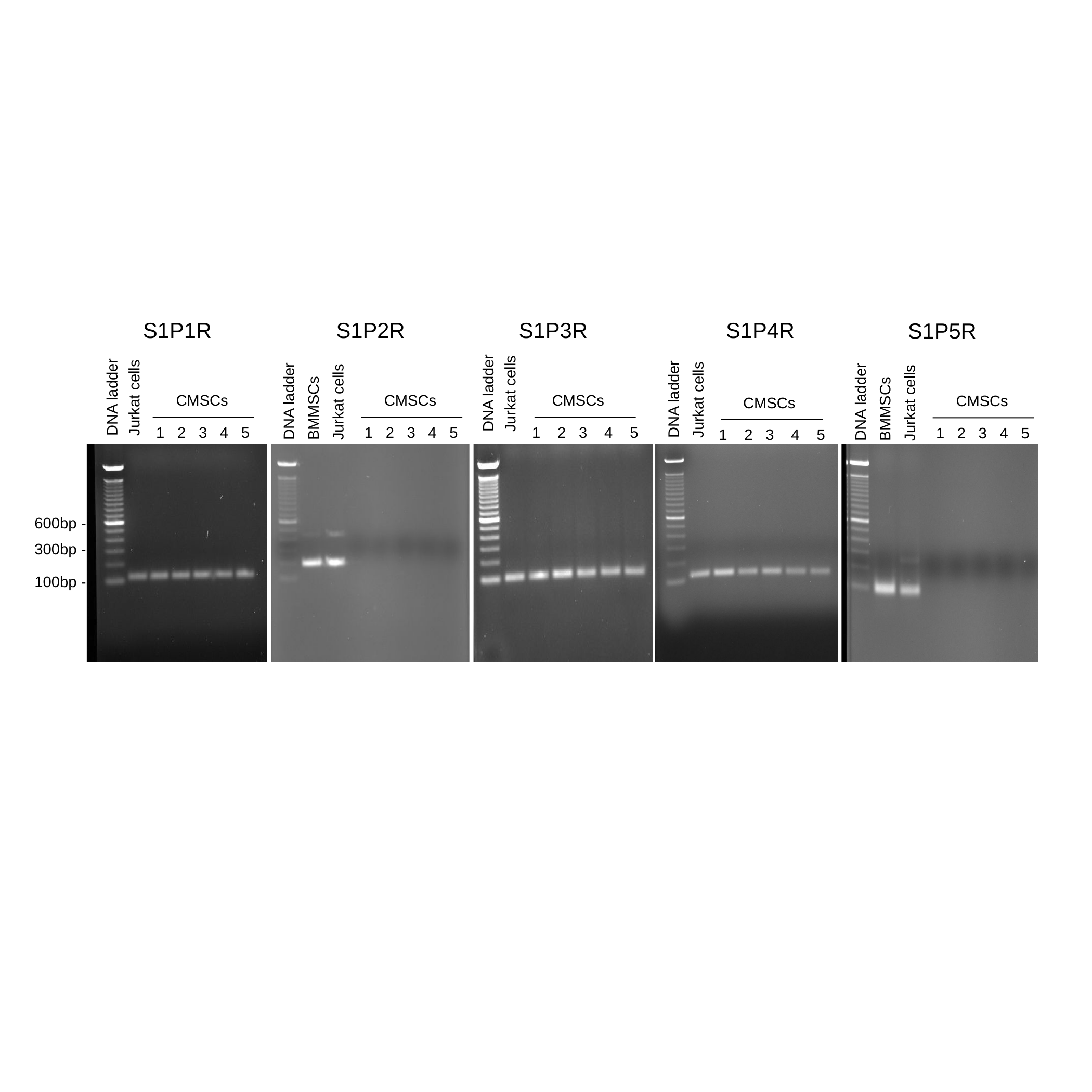

S1P1R
S1P2R
S1P3R
S1P4R
S1P5R
DNA ladder
BMMSCs
Jurkat cells
DNA ladder
BMMSCs
Jurkat cells
DNA ladder
Jurkat cells
DNA ladder
Jurkat cells
DNA ladder
Jurkat cells
CMSCs
CMSCs
CMSCs
CMSCs
CMSCs
1 2 3 4 5
1 2 3 4 5
1 2 3 4 5
1 2 3 4 5
1 2 3 4 5
600bp -
300bp -
100bp -
