# Supplementary material for: Pleiotropic effects of sphingosine-1-phosphate signaling to control human chorionic mesenchymal stem cell physiology
Source: Cell Death Dis. 2017 Jul 13;8(7):e2930–. doi: 10.1038/cddis.2017.312 (PMC5550859; doi:10.1038/cddis.2017.312)
Supplement: Supplementary Figure S2 [file cddis2017312x2.ppt]

## Slide 1
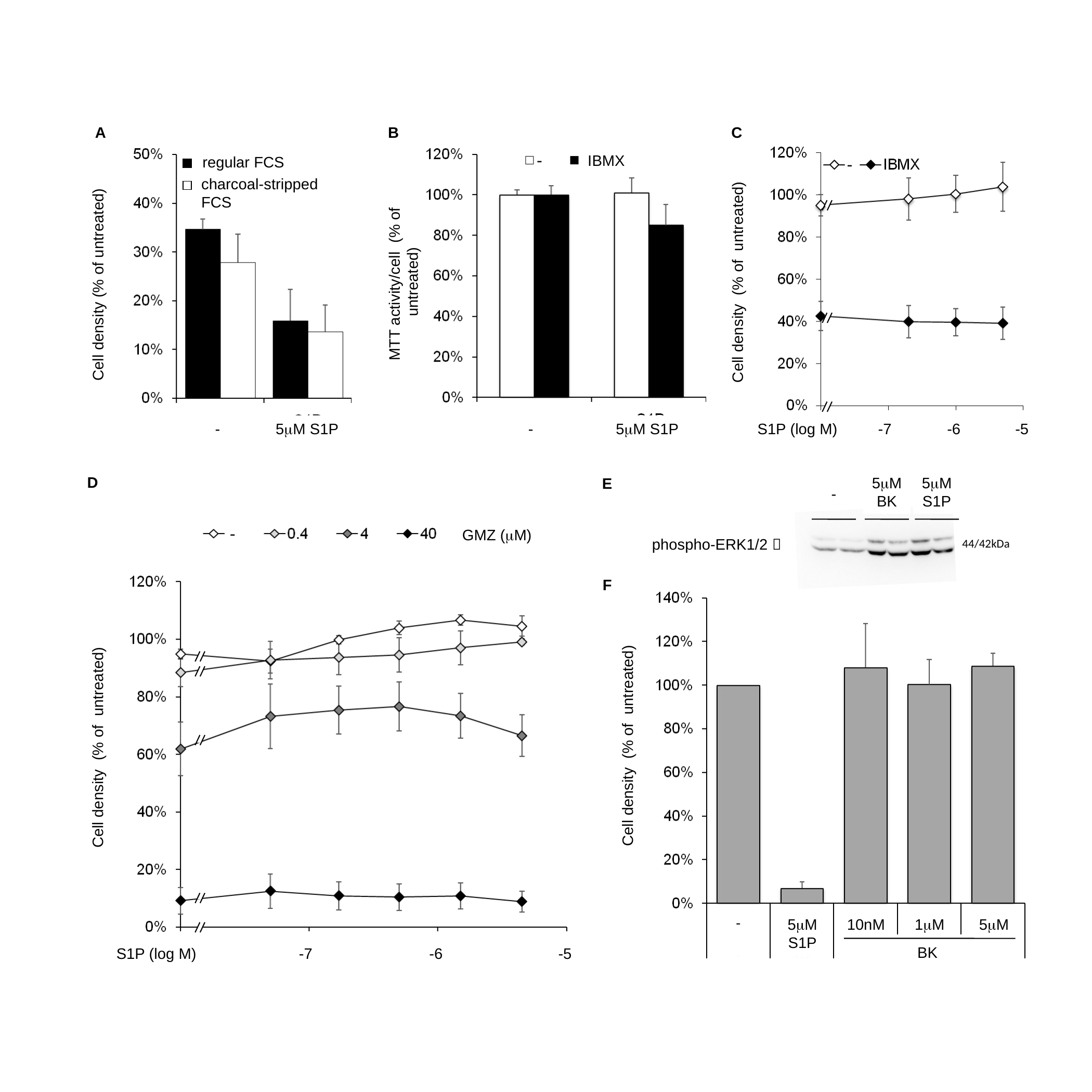

A
B
C
IBMX
regular FCS
charcoal-stripped
FCS
MTT activity/cell (% of untreated)
Cell density (% of untreated)
Cell density (% of untreated)
-
5μM S1P
-
5μM S1P
S1P (log M)
-7
-6
-5
D
5μM BK
5μM
S1P
E
-
GMZ (μM)
phospho-ERK1/2 
44/42kDa
F
Cell density (% of untreated)
Cell density (% of untreated)
-
10nM
1μM
5μM
5μM
S1P
BK
S1P (log M)
-7
-6
-5
