# Supplementary material for: Pleiotropic effects of sphingosine-1-phosphate signaling to control human chorionic mesenchymal stem cell physiology
Source: Cell Death Dis. 2017 Jul 13;8(7):e2930–. doi: 10.1038/cddis.2017.312 (PMC5550859; doi:10.1038/cddis.2017.312)
Supplement: Supplementary Figure S3 [file cddis2017312x3.ppt]

## Slide 1
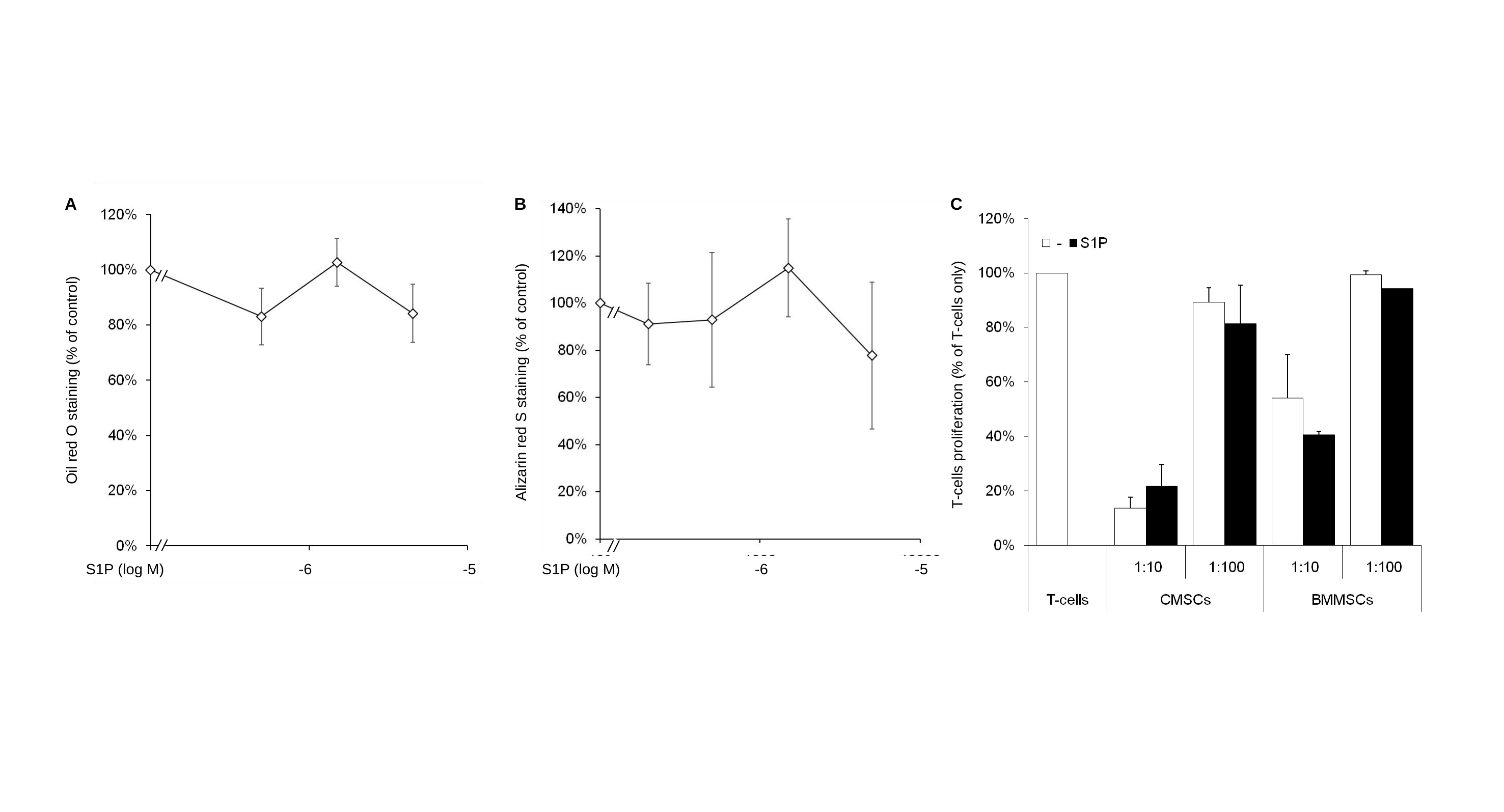

A
B
C
 Oil red O staining (% of control)
Alizarin red S staining (% of control)
T-cells proliferation (% of T-cells only)
 S1P (log M) -6 -5
 S1P (log M) -6 -5
