# Supplementary material for: Pleiotropic effects of sphingosine-1-phosphate signaling to control human chorionic mesenchymal stem cell physiology
Source: Cell Death Dis. 2017 Jul 13;8(7):e2930–. doi: 10.1038/cddis.2017.312 (PMC5550859; doi:10.1038/cddis.2017.312)
Supplement: Supplementary Figure S4 [file cddis2017312x4.ppt]

## Slide 1
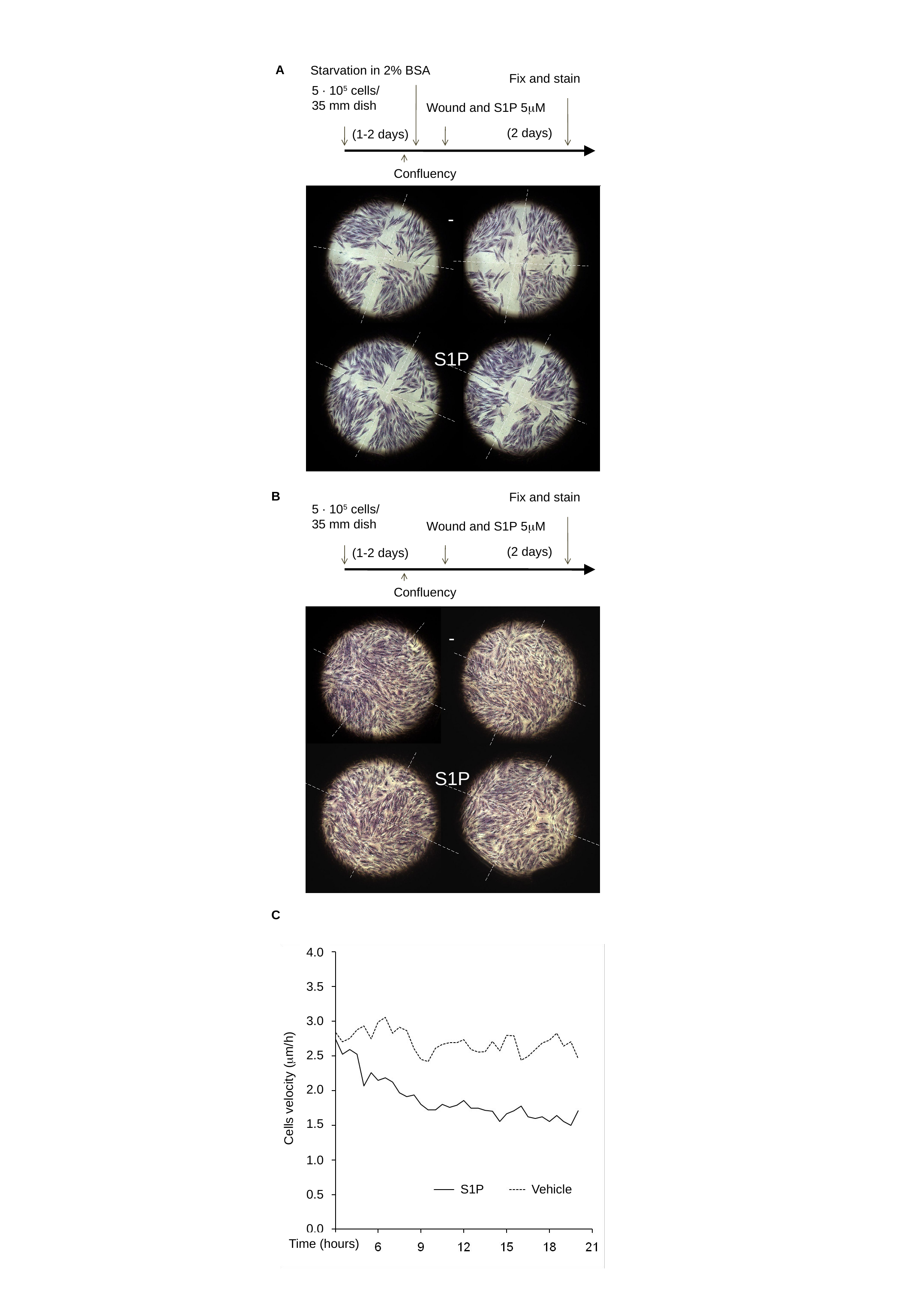

A
Starvation in 2% BSA
Fix and stain
5 ∙ 105 cells/
35 mm dish
Wound and S1P 5μM
(2 days)
(1-2 days)
Confluency
-
S1P
B
Fix and stain
5 ∙ 105 cells/
35 mm dish
Wound and S1P 5μM
(2 days)
(1-2 days)
Confluency
-
S1P
C
4.0
3.5
3.0
2.5
2.0
1.5
1.0
0.5
0.0
Cells velocity (μm/h)
S1P
Vehicle
Time (hours)
