# Supplementary material for: Pleiotropic effects of sphingosine-1-phosphate signaling to control human chorionic mesenchymal stem cell physiology
Source: Cell Death Dis. 2017 Jul 13;8(7):e2930–. doi: 10.1038/cddis.2017.312 (PMC5550859; doi:10.1038/cddis.2017.312)
Supplement: Supplementary Table S1 [file cddis2017312x5.docx]

| **Molecule** | **Affinity**  **(-log10(M))** | **GPCR Specificity** | **Activity** | **Unit** | **Reference** |
| --- | --- | --- | --- | --- | --- |
| Isoproterenol | 5.1-7.0 | β_1,2,3_ adrenosceptor | Full agonist | pKi | 32 |
| Endothelin 1 | 7.8-8.5 | ET_A_ receptor | Full agonist | pIC_50_ | 32 |
| ATP | 5.4-7.8 | P2Y_1,2,3, 11, 13_ recept. | Partial/Full agonist | pIC_50,_ pEC_50_ | 32 |
| Charbacol | 3.2-5.7 | M_1-5_ receptors | Full agonist | pKi | 32 |
| Bradykinin | 4.0-9.3 | B1,2 receptors | Full agonist | pKi, pIC_50_ | 32 |
| S1P | 7.1-9.8 | S1PRs_1,2,3,4,5_ | Agonist | pKd, pEC_50_ | 32 |
| FTY720-P | 5.7-9.5 | S1PRs_1,3,4,5_ | Agonist | pIC_50_ | 33 |
| SEW2871 | 5.5-7.7 | S1PR_1_ | Agonist | pKi, pIC_50_ | 34 |
| JTE-013 | 7.2-7.8 | S1PR_2_ | Antagonist | pIC_50_ | 35 |
| CYM50179 | 7.3 | S1PR_4_ | Agonist | pIC_50_ | 36 |

**Table S1 – *GPCR agonists used to stimulate CMSCs.***

A description of the selectivity of the ligands used for the initial screening of GPCRs expressed by CMSCs and later for the pharmacological characterization of S1P receptors.
