# Supplementary material for: Pleiotropic effects of sphingosine-1-phosphate signaling to control human chorionic mesenchymal stem cell physiology
Source: Cell Death Dis. 2017 Jul 13;8(7):e2930–. doi: 10.1038/cddis.2017.312 (PMC5550859; doi:10.1038/cddis.2017.312)
Supplement: Supplementary Legends [file cddis2017312x8.docx]

**Supplemental figures**

**Figure S1 – *S1PRs expression in CMSCs.***

RT-PCR products quantified in fig. 3 were resolved in a 0.8% agarose gel. The combination of cDNA templates and S1PRs probes are indicated.

**Figure S2 – *Specificity of S1P effect on CMSCs density.***

**(A)** The effect of S1P combined to 1mM IBMX on CMSCs density was assessed comparing charcoal-stripped to regular FCS, n=3. **(B)** Cell proliferation rate was monitored by MTT assay. The signal was measured 24 hours after the addition of 1mM IBMX and S1P and before the toxic effect would produce a marked (>30%) reduction in cell density. Regardless, MTT activity was normalized by the cell number, n=3. **(C)** The effect of S1P combined to 1mM IBMX was tested on BMMSCs density, No statistically significant effect was found, as determined by Kruskal-Wallis test (p=0.75), n=6. **(D)** The effect of S1P treatment combined to the pro-apoptotic agent gemcitabine (GMZ, administered as indicated) was tested at concentrations ranging from not effective (0.4μM) to highly toxic (40μM). No statistically significant effect of S1P was found, as determined by Kruskal-Wallis test (0.4μM p=0.71, 4µM p=0.86, 40μM p=0.99), n=5. **(E)** Bradykinin (BK) induced ERK1/2 phosphorylation as assessed by western blot. **(F)** The effect of BK combined to 1mM IBMX on cell density was assessed finding no statistically significant differences between group averages (control and BK treated) as determined by one-way ANOVA (F(3,12) = 0.19, p = 0.90), n=4.

**Figure S3 – *Functional consequences of S1P signaling on CMSCs differentiation.***

**(A)** CMSCs were differentiated in adipocytes while exposed to increasing concentrations of S1P as indicated. At the end, cells were fixed, stained with Oil red O as in fig.1d, imaged and digitally quantified, n=5. **(B)** CMSCs were differentiated in osteoblasts while exposed to increasing concentrations of S1P as indicated. At the end, cells were fixed, stained with Alizarin red S as in fig.1d, imaged and digitally quantified, n=4. **(C)** To assess the effect of S1P on the immunomodulatory properties of CMSCs and BMMSCs, cultured MSCs were treated for 48 h with S1P or vehicle prior addition of sorted-T cells. MSCs:T-cells ratios were carried out at 1:10 and 1:100. CFSE fluorescence of activated T-cells was analyzed by flow cytometry after 6 days as described in materials and methods, n=3.

**Figure S4 – *Functional consequences of S1P signaling on CMSCs motility.***

Representative fields of wound-healing assay analogous to fig. 8 performed **(A)** in the absence or **(B)** in the presence of 10% FCS, n=3. Seeding and treatments are summarized in the scheme above the micrographs. **(C)** CMSCs were stimulated with 5µM S1P or vehicle. Cell velocity is plotted against a 20-hours time period.

**Video S5 – *Functional consequences of S1P signaling on CMSCs motility.***

Movies corresponding to the wound-healing assay shown in fig.8. **(A)** Untreated cells. **(B)** S1P treated cells.
